# Tighter αC-helix–αL16-helix interactions seem to make p38α less prone to activation by autophosphorylation than Hog1

**DOI:** 10.1042/BSR20160020

**Published:** 2016-04-27

**Authors:** Masha Tesker, Sadiduddin Edbe Selamat, Jonah Beenstock, Ruchama Hayouka, Oded Livnah, David Engelberg

**Affiliations:** * Department of Biological Chemistry, The Alexander Silberman Institute of Life Sciences, The Hebrew University of Jerusalem, Jerusalem 91904, Israel; † Department of Microbiology and Immunology, Yong Loo Lin School of Medicine, National University of Singapore, Singapore 117456, Singapore; ‡ CREATE-NUS-HUJ Cellular and Molecular Mechanisms of Inflammation Programme, National University of Singapore, 1 Create Way, Innovation Wing, #03-09, Singapore 138602, Singapore; § The Wolfson Centre for Applied Structural Biology, The Hebrew University of Jerusalem, Jerusalem 91904, Israel

**Keywords:** autophosphorylation, Hog1, hydrophobic core, kinase, MAP kinase, p38

## Abstract

Many eukaryotic protein kinases (EPKs) are autoactivated through autophosphorylation of their activation loop. Mitogen-activated protein (MAP) kinases do not autophosphorylate spontaneously; relying instead upon mitogen-activated protein kinase (MAPK) kinases (MKKs) for their activation loop phosphorylation. Yet, in previous studies we identified mutations in the yeast MAPK high osmolarity glycerol (Hog1) that render it capable of spontaneous autophosphorylation and consequently intrinsically active (MKK-independent). Four of the mutations occurred in hydrophobic residues, residing in the αC-helix, which is conserved in all EPKs, and in the αL16-helix that is unique to MAPKs. These four residues interact together forming a structural element termed ‘hydrophobic core’. A similar element exists in the Hog1’s mammalian orthologues p38s. Here we show that the ‘hydrophobic core’ is a loose suppressor of Hog1’s autophosphorylation. We inserted 18 point mutations into this core, 17 of which were able to render Hog1 MKK-independent. In p38s, however, only a very few mutations in the equivalent residues rendered these proteins intrinsically active. Structural analysis revealed that a salt bridge between the αC-helix and the αL16-helix that exists in p38α may not exist in Hog1. This bond further stabilizes the ‘hydrophobic core’ of p38, making p38 less prone to de-repressing its concealed autophosphorylation. Mutating equivalent hydrophobic residues in Jnk1 and Erk2 has no effect on their autophosphorylation. We propose that specific structural elements developed in the course of evolution to suppress spontaneous autophosphorylation of Hog1/p38. The suppressors were kept wobbly, probably to allow activation by induced autophosphorylation, but became stricter in mammalian p38s than in the yeast Hog1.

## Abstract (precis)

A structural element termed ‘hydrophobic core’ is a suppressor of spontaneous autophosphorylation in Hog1 and p38s. Practically any mutation in this core of Hog1, but not of p38, evokes spontaneous autophosphorylation. This inherent autophosphorylation suppressor is tighter in mammalian's p38s.

## INTRODUCTION

**Figure 1:**
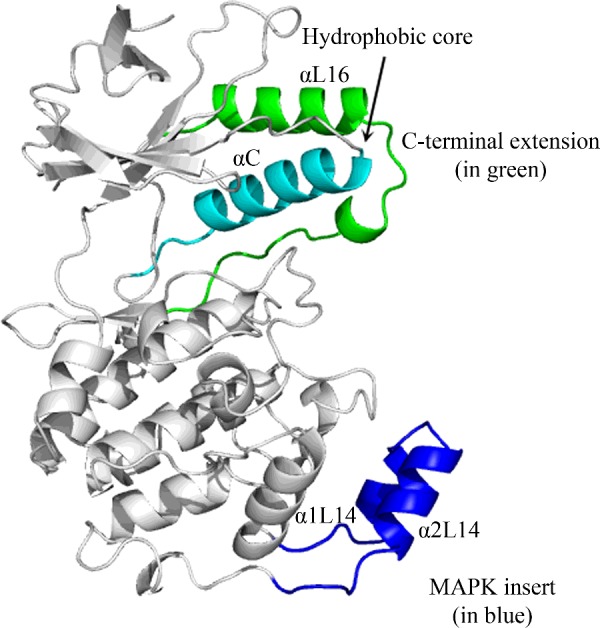
The ‘hydrophobic core’ is formed by interaction between the L16 region and the αC-helix Shown is the crystal structure of p38α (PDB 1P38; grey backbone). The residues forming the ‘hydrophobic core’ reside within the MAPK-specific L16 region (or C-terminal extension, shown in green) and the αC-helix (shown in cyan). Another MAPK-specific domain, known as the MKI, is shown in blue.

Most eukaryotic protein kinases (EPKs) interconvert between active and inactive conformations [1,2]. Although stabilization of the active conformation could be induced by various mechanisms, some specific to each EPK (e.g. allosteric effectors, dimerization or protein–protein interactions) [3,4], the major event that stabilizes the active conformation in many EPKs is the phosphorylation of a particular threonine residue at the activation loop [5–7]. Phosphorylated activation loop threonine forms a network of interactions with various residues, including important interactions with residues of the conserved αC-helix. The conformational changes imposed by activation loop pThr–αC-helix interactions are critical for stabilizing the ATP-binding site, integrity of the R and C spines and the catalytic site [7]. In many EPKs activation loop phosphorylation is acquired via spontaneous autophosphorylation [7–9]. Some sub-families of EPKs, such as the cyclin-dependent kinases (CDKs) and the mitogen-activated protein (MAP) kinases (ERKs, p38s, JNKs and BMK/ERK5), do not commonly autophosphorylate, and so their activation loop phosphorylation must be catalysed by dedicated kinases [e.g. CDK-activated kinase (CAK) and mitogen-activated protein kinase (MAPK) kinases (or MKKs)] [10–14]. Activation loop phosphorylation of MAPKs is also unique in that it occurs not only on the conserved Thr, but also on a neighbouring Tyr residue, part of a TXY motif [12]. Finally, although MAPKs are structurally very similar to all other EPKs, they contain two specific domains known as the MAPK insert (MKI) and the C-terminal extension (L16) ([15], Figure 1).

The autophosphorylation reaction of kinases is only partially understood at the mechanistic and structural levels. It is considered a ‘catch-22’ problem because in order to catalyse autophosphorylation at the activation loop the kinase must possess catalytic properties. However, the kinase is not catalytically active unless phosphorylated on the activation loop [9]. As the mechanism of autophosphorylation of all EPKs is still enigmatic, it is obviously not known why MAPKs do not autophosphorylate spontaneously.

Although autophosphorylation does not occur spontaneously in MAPKs, it seems that they do possess the capability to autophosphorylate. This notion is based on several observations. First, p38β, an isoform of the mammalian p38 family, and JNK2α2, a JNK2 splicing variant, exhibit a noteworthy degree of spontaneous autophosphorylation [16,17]. Second, autophosphorylation of p38α, another isoform of the p38 family, can be induced to autophosphorylate *in vitro* and *in vivo* by direct interaction with TGFβ-activated protein 1 (TAK1)-binding protein (TAB1) [18–20]. Third, p38α and p38β are activated in T cells by autophosphorylation of their activation loop threonine, which is induced by ZAP70-mediated phosphorylation of their Tyr^323^ [21,22]. Tyr^323^ resides in the C-terminal L16 loop region [22]. Fourth, the yeast Erk's orthologue Fus3 is induced to autophosphorylate by interaction with the scaffold protein Ste5 [23]. Finally, all isoforms of p38 and Erk were shown to be capable of autophosphorylation when contain particular point mutations [24–31].

Thus, although MAPKs do not demonstrate a spontaneous autophosphorylation capability, they do possess an autophosphorylation capability that is somehow occluded and could be de-repressed in various manners. MAPKs may serve therefore as an advantageous experimental system for studying the autophosphorylation reaction. The premise is that understanding the mechanisms that block spontaneous autophosphorylation in MAPKs may assist in illuminating the enigmatic mechanism of autophosphorylation in general. In recent studies, therefore, we looked for the structural elements that obstruct autophosphorylation in MAPKs. Several motifs have been identified so far. First, a short region (13 residues), composed of residues from the αG-helix and the MKI, was found to be responsible for suppressing autophosphorylation in p38α. This fragment is slightly different in p38β, sufficient to allow spontaneous autophosphorylation in this isoform [16]. Swapping the region between p38α and p38β provides one with the properties of the other [16]. It is not yet known how this fragment functions. Second, in the yeast p38’s orthologue high osmolarity glycerol (Hog1), the long C-terminal tail is an autophosphorylation inhibitory domain as proposed by the fact that shortening it renders this MAPK spontaneously active (i.e. independent of the relevant MKK) [32]. In this study we tested whether a third structural element, termed the ‘hydrophobic core’, which is very similar and highly conserved in all p38 molecules ranging from yeast to human, also suppresses autophosphorylation [26,33–35]. This element is formed by hydrophobic interactions between the L16 residues Tyr^323^, Phe^327^ and Trp^337^ and the αC-helix's Tyr^69^ (p38α numeration). The possibility that this motif may be involved in controlling autophosphorylation was raised by the observation that in Hog1 some mutations in the residues that form the motif induced autophosphorylation [33–35].

**Figure 2:**
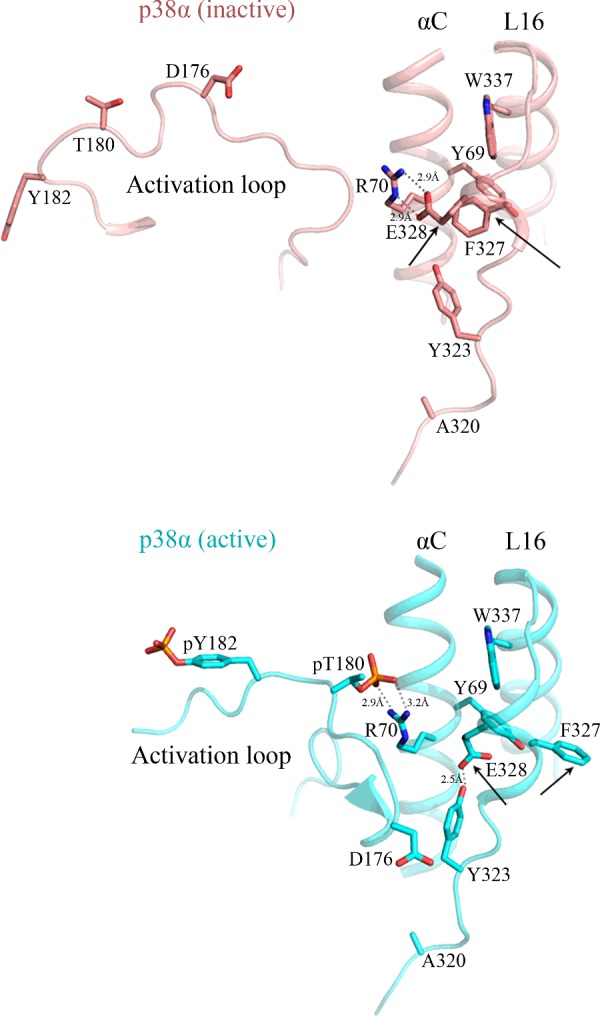
Dramatic changes occur in the ‘hydrophobic core’ and the phosphorylation lip of p38α upon phosphorylation of the TGY motif The crystal structure of the non-phosphorylated form of p38α (PDB 1P38; pink backbone) and the dually phosphorylated p38α (PDB 3PY3; cyan backbone) were aligned according to the αC-helix region. Note the change in orientation of Phe^327^ and Glu^328^ residues (marked by arrows).

Hog1 and its MKK Pbs2 are essential for cell proliferation under osmotic pressure [36,37]. Since Hog1 activity is absolutely dependent on its MKK Pbs2, cells lacking the *HOG1* or the *PBS2* genes cannot proliferate under osmotic pressure [37]. However, Hog1 molecules carrying any of the point mutations Y68H, F318L, F318S, F322L or W332R allow *pbs2∆* cells to proliferate under osmostress ([33–35], Table 1), suggesting that they are intrinsically active and do not require activation by MKK. These Hog1 mutants are in fact phosphorylated in *pbs2∆* cells [33–35]. Insertion of a kinase-dead mutation to them eliminates the phosphorylation and their ability to rescue *pbs2∆* cells [32], strongly suggesting that they bypassed the requirement of MKK-mediated activation because they acquired an efficient autophosphorylation capability [32,35]. In the human p38α, two mutations in an equivalent hydrophobic residue, F327L and F327S, rendered the kinase capable of efficient spontaneous autophosphorylation [26,29]. Some mutations in Tyr^323^ (equivalent to Phe^318^ of Hog1) also rendered p38α spontaneously active, albeit weakly [30]. Mutating Tyr^69^ to His or Trp^337^ to Arg did not have any effect on p38α activity [(26,29] and see below). Later studies have suggested that the interactions between the αC-helix and the αL16-helix are involved in MKK-mediated activity too. Hydrogen exchange studies revealed strong changes in deuterium exchange in Phe^327^ following dual phosphorylation of p38α [38], and the crystal structure of dually phosphorylated p38α showed that Phe^327^ is significantly re-oriented following p38α dual phosphorylation and activation [(39]; Figure 2). In fact, the position of Phe^327^ in the crystal structure of the dually phosphorylated p38α is similar to the position of Ser^327^ and Leu^327^ in the intrinsically active molecules p38α^F327S^ and p38α^F327L^ [39,40]. These observations combined point to the importance of the ‘hydrophobic core’ in regulating both the autophosphorylation activity and the MKK-mediated activity of Hog1 and p38.

**Table 1:** Point mutations found to render Hog1 intrinsically active (Pbs2-independent) and their equivalents in other MAPKs Mutations proved to render the respective kinase intrinsically active are in bold letters. *Mutants showed activity *in vitro*, but not in cell culture. ^#^Mutants showed partial activity *in vitro*.

Hog1	p38α	p38β	p38γ	p38δ	ERK2	References
**Y68Ha**	Y69H^b,c^					a[34], ^b^[26], ^c^[29]
**D170Aa**	**D176A^b,c^**	**D176A^c,d^**	**D179A^b,c,d^**	**D176A^c, d^**	D173Ae	a[34], ^b^[26], ^c^[29], ^d^[28], e[31]
**A314Ta**	A320T^b,c^				A323Te	a[34], ^b^[26], ^c^[29], e[31]
**F318La**	Y323L^*,d^	Y323L^*,c,d^		F324L ^d^	F327Le	a[34], ^b^[26], ^c^[29], ^d^[28], e[31], ^f^[30]
**F318Sa**	Y323S^*,d^	Y323S^c,d^		**F324S^c,d^**	F327Se	
	Y323A^*,f^,					
	Y323D^*,f^,					
	Y323R^*,f^,					
	Y323T^*,f^,					
	Y323Q^*,f^					
	Y323H^*,#,f^,					
	Y323C^*,#,f^,					
	Y323K^*,#,f^,					
	Y323M^*,#,f^,					
	Y323N^*,#,f^,					
	Y323F^*,f^,					
	Y323W^*,f^					
**W320Ra**						a[34]
**F322La**	**F327L^b,c^**	V327L^d^	F330S^b,c^	L328S^c,d^		a[34], ^b^[26], ^c^[29], ^d^[28]
	**F327S^b,c^**	V327S^*,c,d^				
**W332Ra**	W337R^b,c^			W338R^c,d^		a[34], ^b^[26], ^c^[29], ^d^[28]
**N391Da**						a[34]

Notably, yet another point mutation that renders Hog1 and p38s intrinsically active (D170A and D176A respectively) is not involved in the αC-helix–L16 hydrophobic interaction and occurs in the activation loop [26,34].

It was proposed that the hydrophobic interactions between αC-helix and L16 restrain the formation of interactions of Thr^180^ with residues of the αC-helix (particularly Arg^70^) and that disruption of those hydrophobic interactions therefore partially mimics the effect of dual phosphorylation and releases αC-helix for other interactions, including with Thr^180^ [38]. This proposal implies that any disruption of the hydrophobic cluster would be sufficient for activation. In other words, it suggests that there is no specific, rigid, defined conformation that supports autoactivation of Hog1 and p38. Rather, there exists a conformation that suppresses their autophosphorylation activity and any modification that loosens these structural restrictions would trigger autoactivation, regardless of the precise conformation acquired. In this study we tested this proposal rigorously, by mutating Tyr^68^, Phe^318^, Phe^322^ and Trp^332^ of Hog1 to a large variety of residues. We found that almost all mutations rendered Hog1 intrinsically active. This lends strong support to the notion that the αC-helix–αL16-helix hydrophobic interactions function as an inherent ‘locker’, constraining autophosphorylation. We then wondered why in p38α only a few mutations in the ‘hydrophobic core’ rendered it intrinsically active. Through structural modelling of Hog1 we found that the salt bridge between αC-helix and αL16-helix, which occupies Arg^70^ in p38α, probably does not exist in Hog1. This, in our opinion, explains why Hog1 is more permissive for activation by mutations in this element.

## MATERIALS AND METHODS

### Yeast strains and media

The *S. cerevisiae* strains used were: JBY13 (*MAT*a, *ura3-52, lys2-801^amber^, ade2-101^ochre^, trp1-Δ63, his3-Δ200, leu2-Δ1, hog1Δ::TRP1*; obtained from M.C. Gustin, Rice University) and *hog1Δpbs2Δ* strain (*MAT*a, *ura3-52, lys2-801^amber^, ade2-101^ochre^, trp1-Δ63, his3-Δ200, leu2-Δ1, hog1Δ::TRP1, pbs2Δ2*::*LEU2*; [32]). Cultures were maintained on YPD (1% yeast extract, 2% Bacto-Peptone, 2% glucose) or on the synthetic medium YNB(-URA) (0.17% yeast nitrogen base without amino acid and (NH_4_)_2_SO_4_, 0.5% ammonium sulfate, 2% glucose and 40 mg/l of the required nutrients). To induce osmotic stress we used YPD supplemented with 1 M NaCl.

### Plasmids constructions and mutagenesis procedures

Site-directed mutagenesis was performed according to the manufacturer's instructions, using the PfuUltraII Fusion HotStart DNA Polymerase (Agilent # 600670) and pBluescriptIISK+ containing the relevant DNA as a template. Primers used are listed in Table 2. Then, the DNA was cloned to the vector of interest, using the BstEII (NEB # R3162S) and BlpI (NEB # R0585S) restriction enzymes for *HOG1* and the EcoRI (NEB #R3101S) restriction enzyme for p38α. All *HOG1* molecules were expressed from the pES86 vector as N-terminal 3X-HemeAgluttinin (3XHA)-tagged proteins, as described previously [35]. Native *HOG1* and various derivatives were sub-cloned between the *ADH1* promoter and terminator. The pES86 vector further harbours the *URA3* gene and the 2μ-element. For mammalian expression, the pcDNA3 (Startagene) and pBabe (Addgene) vectors were used with the ORFs tagged N-terminally with 3XHA. For bacterial expression, the pET15b vector was used, with the ORFs hexahistidine-tagged N-terminally, as described previously [26,28,29].

**Table 2:** Primers used in polymerase chain reaction and site-directed mutagenesis reactions

Primer name	Primer sequence
HOG1 F318A Forward	5′- CAGTAGCCGATGCCAAGGCCGATTGGCACTTTAATG -3′
HOG1 F318A Reverse	5′- CATTAAAGTGCCAATCGGCCTTGGCATCGGCTACTG -3′
HOG1 F318P Forward	5′- CAGTAGCCGATGCCAAGCCCGATTGGCACTTTAATG -3′
HOG1 F318P Reverse	5′- CATTAAAGTGCCAATCGGGCTTGGCATCGGCTACTG -3′
HOG1 F318Y Forward	5′- CAGTAGCCGATGCCAAGTACGATTGGCACTTTAATG -3′
HOG1 F318Y Reverse	5′- CATTAAAGTGCCAATCGTACTTGGCATCGGCTACTG -3′
HOG1 F318T Forward	5′- CCAGTAGCCGATGCCAAGACCGATTGGCACTTTAATG -3′
HOG1 F318T Reverse	5′- CATTAAAGTGCCAATCGGTCTTGGCATCGGCTACTGG -3′
HOG1 F318E Forward	5′- CCAGTAGCCGATGCCAAGGAGGATTGGCACTTTAATGACG -3′
HOG1 F318E Reverse	5′- CGTCATTAAAGTGCCAATCCTCCTTGGCATCGGCTACTGG -3′
HOG1 F318R Forward	5′- CCAGTAGCCGATGCCAAGAGGGATTGGCACTTTAATGACG -3′
HOG1 F318R Reverse	5′- CGTCATTAAAGTGCCAATCCCTCTTGGCATCGGCTACTGG -3′
HOG1 F318V Forward	5′- CAGTAGCCGATGCCAAGGTCGATTGGCACTTTAATG -3′
HOG1 F318V Reverse	5′- CATTAAAGTGCCAATCGACCTTGGCATCGGCTACTG -3′
HOG1 Y68A Forward	5′- GCTGGCCAAAAGGACAGCTCGTGAACTAAAACTAC -3′
HOG1 Y68A Reverse	5′- GTAGTTTTAGTTCACGAGCTGTCCTTTTGGCCAGC -3′
HOG1 Y68F Forward	5′- GCTGGCCAAAAGGACATTTCGTGAACTAAAACTAC -3′
HOG1 Y68F Reverse	5′- GTAGTTTTAGTTCACGAAATGTCCTTTTGGCCAGC -3′
HOG1 Y68K Forward	5′- GCTGGCCAAAAGGACAAATCGTGAACTAAAACTAC -3′
HOG1 Y68K Reverse	5′- GTAGTTTTAGTTCACGATTTGTCCTTTTGGCCAGC -3′
HOG1 Y68P Forward	5′- GCTGGCCAAAAGGACACCTCGTGAACTAAAACTAC -3′
HOG1 Y68P Reverse	5′- GTAGTTTTAGTTCACGAGGTGTCCTTTTGGCCAGC -3′
HOG1 D170G Forward	5′- GTCTAGCAAGAATTCAAGGCCCTCAAATGACAGGC -3′
HOG1 D170G Reverse	5′- GCCTGTCATTTGAGGGCCTTGAATTCTTGCTAGAC -3′
HOG1 D170F Forward	5′- GTCTAGCAAGAATTCAATTCCCTCAAATGACAGGC -3′
HOG1 D170F Reverse	5′- GCCTGTCATTTGAGGGAATTGAATTCTTGCTAGAC -3′
HOG1 D170P Forward	5′- GTCTAGCAAGAATTCAACCCCCTCAAATGACAGGC -3′
HOG1 D170P Reverse	5′- GCCTGTCATTTGAGGGGGTTGAATTCTTGCTAGAC -3′
HOG1 D170S Forward	5′- GTCTAGCAAGAATTCAAAGCCCTCAAATGACAGGC -3′
HOG1 D170S Reverse	5′- GCCTGTCATTTGAGGGCTTTGAATTCTTGCTAGAC -3′
HOG1 F322A Forward	5′- CAAGTTCGATTGGCACGCTAATGACGCTGATCTGCC -3′
HOG1 F322A Reverse	5′- GGCAGATCAGCGTCATTAGCGTGCCAATCGAACTTG -3′
HOG1 F322W Forward	5′- CAAGTTCGATTGGCACTGGAATGACGCTGATCTGCC -3′
HOG1 F322W Reverse	5′- GGCAGATCAGCGTCATTCCAGTGCCAATCGAACTTG -3′
HOG1 F322V Forward	5′- CCAAGTTCGATTGGCACGTTAATGACGCTGATCTGC -3′
HOG1 F322V Reverse	5′- GCAGATCAGCGTCATTAACGTGCCAATCGAACTTGG -3′
HOG1 W332A Forward	5′- GATCTGCCTGTCGATACCGCGCGTGTTATGATGTACTC -3′
HOG1 W332A Reverse	5′- GAGTACATCATAACACGCGCGGTATCGACAGGCAGATC -3′
HOG1 W332F Forward	5′- GATCTGCCTGTCGATACCTTTCGTGTTATGATGTACTC -3′
HOG1 W332F Reverse	5′- GAGTACATCATAACACGAAAGGTATCGACAGGCAGATC -3′
HOG1 W332L Forward	5′- GATCTGCCTGTCGATACCCTGCGTGTTATGATGTACTC -3′
HOG1 W332L Reverse	5′- GAGTACATCATAACACGCAGGGTATCGACAGGCAGATC -3′
HOG1 W332S Forward	5′- GATCTGCCTGTCGATACCTCGCGTGTTATGATGTACTC -3′
HOG1 W332S Reverse	5′- GAGTACATCATAACACGCGAGGTATCGACAGGCAGATC -3′
HOG1 N323E Forward	5′- GTTCGATTGGCACTTTGAGGACGCTGATCTGCCTG -3′
HOG1 N323E Reverse	5′- CAGGCAGATCAGCGTCCTCAAAGTGCCAATCGAAC -3′
HOG1 N323A Forward	5′- GTTCGATTGGCACTTTGCTGACGCTGATCTGCCTG -3′
HOG1 N323A Reverse	5′- CAGGCAGATCAGCGTCAGCAAAGTGCCAATCGAAC -3′
p38α F327V Forward	5′- CCTTATGATCAGTCCGTTGAAAGCAGGGACC -3′
p38α F327V Reverse	5′- GGTCCCTGCTTTCAACGGACTGATCATAAGG -3′
p38α E328N Forward	5′- GATCCTTATGATCAGTCCTTTAACAGCAGGGACCTCCTTATAG -3′
p38α E328N Reverse	5′- CTATAAGGAGGTCCCTGCTGTTAAAGGACTGATCATAAGGATC -3′
p38α E328A Forward	5′- GATCAGTCCTTTGCAAGCAGGGACCTCC -3′
p38α E328A Reverse	5′- GGAGGTCCCTGCTTGCAAAGGACTGATC -3′
p38β V327F Forward	5′- GCCATATGATGAGAGCTTTGAGGCCAAGGAGC -3′
p38β V327F Reverse	5′- GCTCCTTGGCCTCAAAGCTCTCATCATATGGC -3′
p38γ Y72H Forward	5′- CTGTTCGCCAAGCGCGCCCACCGCGAGCTGCGCCTGCTC -3′
p38γ Y72H Reverse	5′- GAGCAGGCGCAGCTCGCGGTGGGCGCGCTTGGCGAACAG -3′
p38γ Y326S Forward	5′- GAGCCCCAGGTCCAGAAGTCTGATGACTCCTTTGACGAC -3′
p38γ Y326S Reverse	5′- GTCGTCAAAGGAGTCATCAGACTTCTGGACCTGGGGCTC -3′
p38γ Y326A Forward	5′- GAGCCCCAGGTCCAGAAGGCTGATGACTCCTTTGACGAC -3′
p38γ Y326A Reverse	5′- GTCGTCAAAGGAGTCATCCGACTTCTGGACCTGGGGCTC -3′
p38γ Y326L Forward	5′- GAGCCCCAGGTCCAGAAGCTTGATGACTCCTTTGACGAC -3′
p38γ Y326L Reverse	5′- GTCGTCAAAGGAGTCATCAAGCTTCTGGACCTGGGGCTC -3′
p38γ Y326T Forward	5′- GAGCCCCAGGTCCAGAAGACTGATGACTCCTTTGACGAC -3′
p38γ Y326T Reverse	5′- GTCGTCAAAGGAGTCATCAGTCTTCTGGACCTGGGGCTC -3′
p38γ F330T Forward	5′- CAGAAGTATGATGACTCCACTGACGACGTTGACCGCACAC -3′
p38γ F330T Reverse	5′- GTGTGCGGTCAACGTCGTCAGTGGAGTCATCATACTTCTG -3′
p38γ F330R Forward	5′- CAGAAGTATGATGACTCCCGTGACGACGTTGACCGCACAC -3′
p38γ F330R Reverse	5′- GTGTGCGGTCAACGTCGTCACGGGAGTCATCATACTTCTG -3′
p38γ F330A Forward	5′- CAGAAGTATGATGACTCCGCTGACGACGTTGACCGCACAC -3′
p38γ F330A Reverse	5′- GTGTGCGGTCAACGTCGTCAGCGGAGTCATCATACTTCTG -3′
p38γ F330P Forward	5′- CAGAAGTATGATGACTCCCCTGACGACGTTGACCGCACAC -3′
p38γ F330P Reverse	5′- GTGTGCGGTCAACGTCGTCAGGGGAGTCATCATACTTCTG -3′
p38γ F330L Forward	5′- CAGAAGTATGATGACTCCCTTGACGACGTTGACCGCACAC -3′
p38γ F330L Reverse	5′- GTGTGCGGTCAACGTCGTCAAGGGAGTCATCATACTTCTG -3′
p38δ F324A Forward	5′- GGAGGCCCAGCAGCCGGCTGATGATTCCTTAG -3′
p38δ F324A Reverse	5′- CTAAGGAATCATCAGCCGGCTGCTGGGCCTCC -3′
p38δ F324E Forward	5′- GGAGGCCCAGCAGCCGGAAGATGATTCCTTAG -3′
p38δ F324E Reverse	5′- CTAAGGAATCATCTTCCGGCTGCTGGGCCTCC -3′
p38δ F324P Forward	5′- GGAGGCCCAGCAGCCGCCTGATGATTCCTTAG -3′
p38δ F324P Reverse	5′- CTAAGGAATCATCAGGCGGCTGCTGGGCCTCC -3′
p38δ F324R Forward	5′- GGAGGCCCAGCAGCCGCGTGATGATTCCTTAG -3′
p38δ F324R Reverse	5′- CTAAGGAATCATCACCCGGCTGCTGGGCCTCC -3′
p38δ F324T Forward	5′- GGAGGCCCAGCAGCCGACTGATGATTCCTTAG -3′
p38δ F324T Reverse	5′- CTAAGGAATCATCAGTCGGCTGCTGGGCCTCC -3′
p38δ F324Y Forward	5′- GGAGGCCCAGCAGCCGTATGATGATTCCTTAG -3′
p38δ F324Y Reverse	5′- CTAAGGAATCATCATACGGCTGCTGGGCCTCC -3′
JNK1 Y71H Forward	5′- GAAGCTAAGCCGACCATTTCAGAATCAGACTCATGCCAAGCGGGCC CACAGAGAGCTAG -3′
JNK1 Y71H Reverse	5′- CTAGCTCTCTGTGGGCCCGCTTGGCATGAGTCTGATTCTGAAATGGT CGGCTTAGCTTC -3′
JNK1 I337S Forward	5′- GAAGCTCCACCACCAAAGAGCCCTGACAAGCAGTTAGATG -3′
JNK1 I337S Reverse	5′- CATCTAACTGCTTGTCAGGGCTCTTTGGTGGTGGAGCTTC -3′
JNK1 P338S Forward	5′- GAAGCTCCACCACCAAAGATCTCTGACAAGCAGTTAGATG -3′
JNK1 P338S Reverse	5′- CATCTAACTGCTTGTCAGAGATCTTTGGTGGTGGAGCTTC -3′
JNK1 L341Q Forward	5′- CCAAAGATCCCTGACAAGCAGCAAGATGAAAGGGAACACACAATAG -3′
JNK1 L341Q Reverse	5′- CTATTGTGTGTTCCCTTTCATCTTGCTGCTTGTCAGGGATCTTTGG -3′
JNK1 W351R Forward	5′- GCAGTTAGATGAAAGGGAACACACAATAGAAGAGAGGAAAGAATT GATATATAAGGAAGTTATGG -3′
JNK1 W351R Reverse	5′- CCATAACTTCCTTATATATCAATTCTTTCCTCTCTTCTATTGTGTGTT CCCTTTCATCTAACTGC -3′

### Spot assay

Yeast cultures were grown in a liquid medium YNB(-URA), to mid-logarithmic phase (OD_600_∼0.4). Five decimal dilutions were created (approximately 10^7^, 10^6^, 10^5^, 10^4^ and 10^3^ cells/ml) and 5 μl from each dilution were plated on YPD plates supplemented with 1 M NaCl. Plates were incubated at 30°C for 3 days. All experiments were performed in at least three independent biological replicates.

### Preparation of protein lysates of yeast cells via the TCA precipitation method

Yeast cultures were grown in a liquid medium (YNB-URA) to a mid-logarithmic phase (OD_600_∼0.5), precipitated by centrifugation (5 min, 2000 × ***g***) and re-suspended in a medium supplemented or not supplemented with 1 M NaCl. At the time points indicated in the relevant experiments a sample of 10 ml was collected and precipitated by centrifugation (5 min, 2000 × ***g***). The yeast pellet was washed with 10 ml of 20% TCA and precipitated again by centrifugation (10 min, 2000 × ***g***). The washed pellet was re-suspended in 200 μl of 20% TCA and 400 mg of glass beads were added. Cells were broken by vortex-mixing for 8 min, and the supernatants were transferred to a clean tube. The beads that were left in the tube were washed twice with 200 μl of 5% TCA and the supernatants were combined in the same tube. The proteins were precipitated by centrifugation (10 min, 1100 × ***g***) and the pellet was re-suspended in 100 μl of Laemmli buffer and 50 μl of 1 M Tris base and boiled at 100°C for 3 min.

### Protein purification

Protein purification from *E. coli* Rosetta strain cells (Novagene) was performed using Ni-NTA beads (Hadar Biotech), as previously described [26]. Protein concentrations were determined by the Bradford method. 100 ng were mixed with X4 Laemmli buffer and boiled at 100°C for 4 min.

### Western blot analysis

Mammalian cell protein lysates were prepared by decanting the growth medium, washing cells with PBS and adding Laemmli buffer directly to the plate. Lysates were then collected and boiled at 100°C for 10 min. Unless specified otherwise, 30 μg of the different lysates were separated via SDS/10% PAGE and transferred to a nitrocellulose membrane. The membranes were incubated with the appropriate antibodies. Antibodies used in this study were: anti-phospho-p38 (cell signalling #9211), anti-HA (from the 12CA5 hybridoma cell line), anti-HOG1 (santa cruz #sc-9079), anti-MAPKAPK2 (cell signalling, #3042) or anti-phospho-MAPKAPK2 (cell signalling #3007). All experiments were performed in at least three independent biological replicates.

### In vitro kinase assay

All reactions with GST-ATF2 or GST-c-Jun as substrates were conducted in 96-well plates in triplicates. To initialize the reaction, 45 μl of the reaction mixture were added to 5 μl of the p38 or c-Jun N-terminal kinase (JNK) enzymes (0.2 μg, 100 nM, of the purified hexahistidine tag-protein). Final reaction conditions were 25 mM Hepes pH 7.5, 20 mM MgCl_2_, 20 mM 2-glycerolphosphate, 0.1 mM Na_3_VO_4_, 1 mM dithiothreitol, 40 μg GST-ATF2, 50 μM ATP (kinase buffer) and 0.5 μCi of [γ-^32^P]ATP. Reaction proceeded for 10 min at 30°C with agitation and was terminated with 50 μl of 0.5 M EDTA pH 8 (250 mM final). Following reaction termination, 15 μl from each set of reactions were subjected to SDS/PAGE, stained with Coomassie, dried and exposed to film. Aliquots of 85 μl from each reaction well were spotted on to 3 cm × 3 cm Whatman 3MM Chromatography paper squares (GE Healthcare #3030917) and air-dried. Each square was rinsed three times with 10% trichloroacetic acid and 3% sodium pyrophosphate (10 ml/square) for 1.5 h (each time) with gentle agitation, and a fourth wash was given overnight without shaking. The following day, the squares were rinsed twice with 100% ethanol (4 ml/square) for 20 min each time and air-dried. The radioactivity of each square was counted using a scintillation counter running a ^32^P Cherenkov program. Autophosphorylation reactions of proteins purified from *E. coli* were conducted under similar conditions, with 1 μg of the tested protein and without GST-ATF2. These reactions were analysed by SDS/PAGE. For activation of p38 with MKK6, an active mutant of MKK6, termed MKK6^EE^, in which Ser^207^ and Thr^211^ were both mutated to Glu, was used under similar conditions. The activation of JNK was performed under similar conditions using the active mutant of MKK7, MKK7^DD^, in which Ser^271^ and Thr^275^ were mutated to Asp. All experiments were performed in three technical replicates in at least two independent biological replicates.

### Mammalian cell culture

HEK293 cells were grown in Dulbecco's MEM supplemented with 10% fetal bovine serum, Na-pyruvate and antibiotics. Cells were grown at 37°C and 5% CO_2_. Cells were transfected with the calcium phosphate method.

### Luciferase assay

7.5×10^5^ HEK293T cells were co-transfected with p38α expressing vector, firefly and *Renilla* luciferase vectors. The assay was preformed 48 h post-transfection. Cells were lysed in accordance with the manufacturer's protocol (Dual-Luciferase® Reporter Assay System, Promega, #E1910) and the luminescence was measured with a GloMax® 20/20 Single Tube Luminometer (Promega). The firefly luciferase reporter activity was normalized to the *Renilla* luciferase reaction. All experiments were performed in three technical replicates in three independent biological replicates.

## RESULTS

### Most substitutions of Tyr^68^, Phe^318^, Phe^322^ or Trp^332^ render Hog1 intrinsically active (Pbs2-independent)

**Figure 3:**
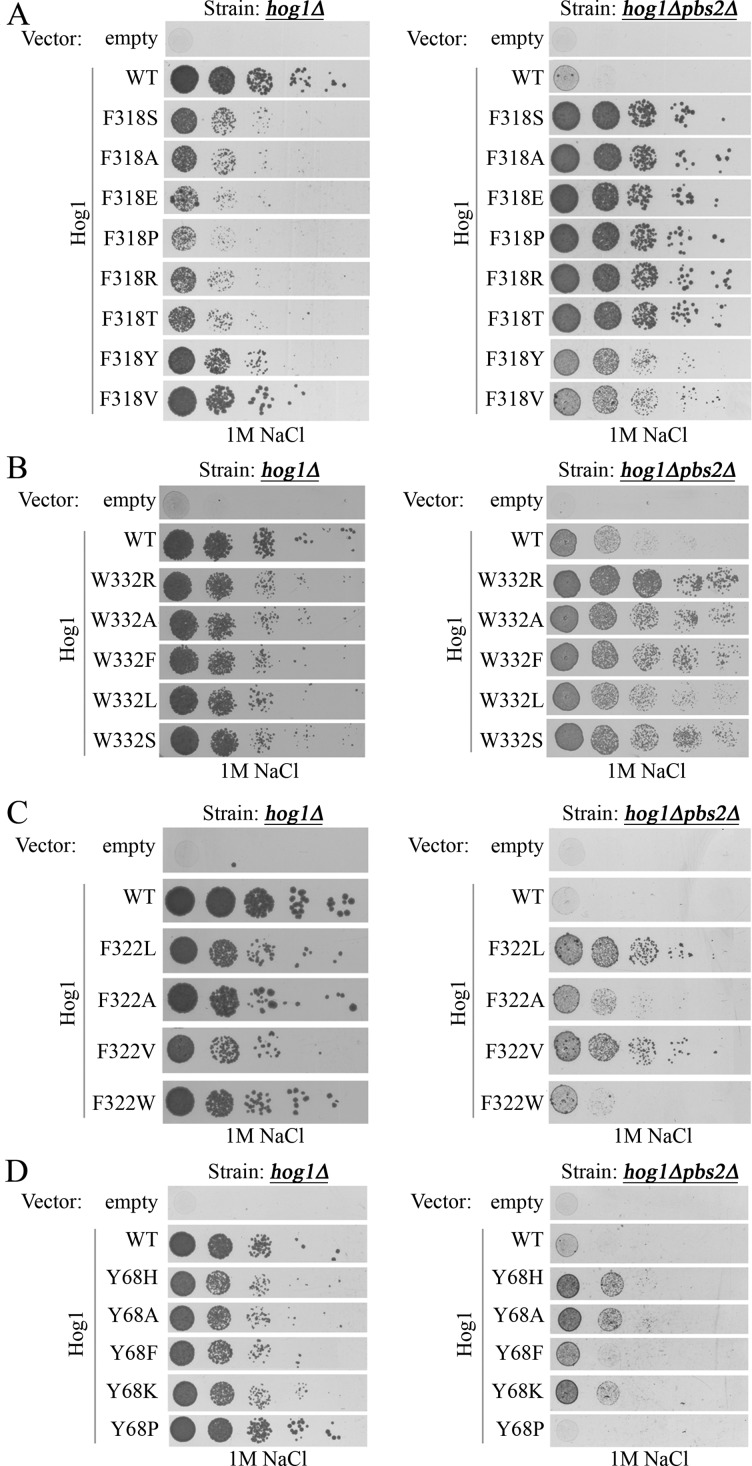
Most mutations that replace residues that form the ‘hydrophobic core’ of Hog1 render the kinase intrinsically active, namely, independent of the MKK Pbs2 *hog1Δ* cells (left panels) and *hog1Δpbs2Δ* cells (right panels), expressing Hog1 molecules with the indicated residues at position 318 (**A**), 332 (**B**), 322 (**C**) or 68 (**D**) were plated in five dilutions on plates containing YPD supplemented with 1 M NaCl.

To address the hypothesis that any modification of the hydrophobic interactions between αC-helix and αL16-helix in Hog1 would lead to autophosphorylation and intrinsic activity, we converted Tyr^68^, Phe^318^, Phe^322^ or Trp^332^ to a variety of other residues (Table 3). To test which of the mutants acquired an intrinsic, Pbs2-independent, activity, we expressed them in *hog1∆pbs2∆* cells and monitored growth of the expressing cells under osmostress (1 M NaCl). Strikingly, replacing Phe^318^ not only with Leu or Ser [34], but with all residues tested (Ala, Glu, Pro, Arg, Thr, Tyr, Val) rendered Hog1 Pbs2-independent (Figure 3A, right panel). Interestingly, replacing Phe^318^ with Tyr or Val was less effective in elevating intrinsic activity compared with all other mutants (Figure 3A, right panel), probably because of the hydrophobic nature of these two residues. Thus, with the exception of a Hog1 protein that carries Phe at position 318 (namely, the wild type protein), Hog1 proteins carrying any of the tested residues at this position are intrinsically active. We then tested Hog1 proteins mutated at position 332 (carrying Arg, Ala, Phe, Leu or Ser, replacing the natural Trp) and found that all were intrinsically active, with Hog1^W332L^ being less efficient than other mutants (Figure 3B, right panel). In addition, replacing Phe^322^ with Leu, Ala, Val or Trp also rendered the kinase Pbs2-independent (Figure 3C, right panel). Hog1^F322L^ and Hog1^F322V^ rescued *hog1∆pbs2∆* cells very efficiently, but Hog1^F322A^ and Hog1^F322W^ allowed only a partial rescue (Figure 3C). Finally, mutations at position 68 (His, Ala, Phe, Lys or Pro, replacing the native Tyr) also rescued *hog1Δpbs2Δ* cells, but only somewhat partially and with different efficiencies, including no rescue at all by Hog1^Y68P^ (Figure 3D, right panel). Notably, Tyr^68^ is part of the αC-helix and an insertion of a proline might break this critical secondary structure.

**Table 3:** Mutations studied in this work Mutations proved to render the respective kinase intrinsically active are in bold letters. **This mutation caused loss of function of the kinase. ^#^Mutants showed partial activity.

	New mutations designed						
Previously tested mutations	Hog1	p38α	p38β	p38γ	p38δ	JNK1	References
**Hog1^Y68H,a^**	**Y68A**			Y72H		Y71H	a[34]
	Y68F^#^						
	**Y68K**						
	Y68P						
**Hog1^F318S,a^**	**F318A**			Y326S Y326A	F324A	I337S	a[34], ^c^[29],
**p38δ^F324S,c,d^**	**F318E**			Y326L Y326T	F324E	P338S	^d^[28]
	**F318P**				F324P		
	**F318R**				F324R		
	**F318T**				F324T		
	**F318Y^#^**				F324Y		
	**F318V^#^**						
**Hog1^F322L,a^**	**F322A^#^**	F327V	V327F	F330T F330R		L342Q	a[34], ^b^[26],
**p38α^F327S,b,c^**	**F322V**			F330A			^c^[29], ^d^[28]
p38β^V327L,d^	F322W^#^			F330P			
p38β^V327S,c,d^				F330L			
p38γ^F330S,b,c^							
**Hog1^W332R,a^**	**W332A**					W352R	a[34]
	**W332F**						
	**W322L**						
	**W322S**						
							
							
**D170Aa**	**D170G**						a[34]
	D170F						
	D170P**						
	**D170S**						
	N323E	E328N					
	N323A	E328A					
	**N323E+Y68H**	N323A+Y68H					
	**E328N+Y69H**	E328A+Y69H					

### Different expression and phosphorylation levels of the Hog1 molecules mutated at the ‘hydrophobic core’

**Figure 4:**
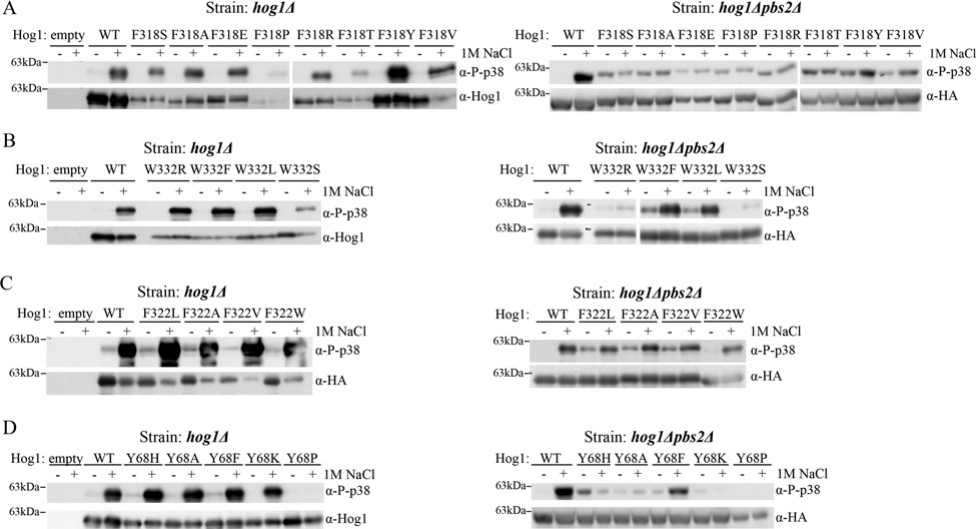
Most of the Hog1 molecules tested are phosphorylated in cells lacking Pbs2 Western blot analysis with the indicated antibodies was performed on protein lysates prepared from *hog1Δ* cells (left panels) or from *hog1Δpbs2Δ* cells (right panels), expressing the indicated Hog1 molecules. Cells were grown to logarithmic phase on YNB(-URA) medium, washed and incubated in YPD supplemented with 1 M NaCl for 10 min. prior to lysis. α-Phospho-p38 antibody was used to determine the phosphorylation of the TGY motif of Hog1. α-HA or α-Hog1 antibodies were used for determination of the steady-state levels of expressed Hog1 proteins.

Previous analysis showed that the intrinsically active mutants bypassed the requirement of Pbs2-dependent phosphorylation by gaining autophosphorylation capability [32,35]. In some cases autophosphorylation is induced in response to osmostress in *pbs2∆* cells, suggesting that there exists an osmostress-induced Pbs2-independent pathway that enhances autophosphorylation of those mutants [32]. It is important therefore to monitor the phosphorylation levels of the new Hog1 mutants expressed in *hog1∆pbs2∆* cells following exposure to osmotic stress. Prior to testing the phosphorylation levels we monitored their steady-state levels. As shown in Figure 4 the levels of proteins mutated in Phe^318^ were low when expressed in *hog1∆* cells, with two exceptions, Hog1^F318Y^ that was expressed at levels similar to those of Hog1^WT^ and Hog1^F318P^ that was expressed at very low levels (Figure 4A, left lower panel). This explains why Hog1^F318P^ fails to rescue *hog1∆* cells from osmostress (Figure 3A, left panel). As all proteins were expressed from identical plasmids, the differences in expression levels are intriguing. Probably, overexpression of the most active mutants is hazardous, as reflected in the slow growth rates of *hog1∆* cells expressing them [(35]; and Figure 3A left panel). It could be that most of the culture cells that overexpress the variants proliferate very slowly (see for example in [35]) and only those that for some reason express lower levels are capable of some growth and are ultimately selected and takeover the culture.

Following exposure to 1 M NaCl, all mutants were phosphorylated. The phosphorylation level, as determined by anti-phospho-p38 antibodies, was correlated with their expression levels (Figure 4A, upper left panel). In *hog1∆pbs2∆* cells all proteins carrying a mutation in Phe^318^, including Hog1^F318P^, were expressed at similar levels and were constitutively phosphorylated, regardless of osmotic stress (Figure 4A, right panel). In these cells the mutants can exhibit only their intrinsic, Pbs2-independent, activity, and therefore, their total activity is lower and less hazardous to cell growth. Proteins carrying various residues in the 332 position were phosphorylated in *hog1∆pbs2∆* cells at different levels (Figure 4B, right panel). Hog1^W332F^ and Hog1^W332L^ were found to be spontaneously phosphorylated in these cells and phosphorylation was further induced in response to exposure to NaCl. Hog1^W332S^ was phosphorylated at very low levels, although it did rescue the cells from osmostress (Figure 3B). Hog1^F322A^ and Hog1^F322V^ were spontaneously phosphorylated in *hog1∆pbs2∆* cells and the phosphorylation was further induced in response to osmostress (Figure 4C, right panel). Correlated with its reduced ability to rescue *hog1∆pbs2∆* cells from osmostress (Figure 3C), spontaneous phosphorylation levels of Hog1^F322W^ were reduced compared with the other two mutants. Finally, the mutants at position 68 were phosphorylated at very low and constitutive levels in *hog1∆pbs2∆* cells, with the exception of Hog1^Y68F^ whose phosphorylation was strongly induced in response to NaCl (Figure 4D). Hog1^Y68K^ was phosphorylated at levels below detection in *hog1∆pbs2∆* cells, although it did rescue the cells from osmostress (Figure 3D). Hog1^Y68P^ seemed to have lost its capability to be phosphorylated even in the presence of Pbs2. This observation could explain its inability to rescue *hog1∆pbs2∆* cells from osmostress (Figure 3D, right panel).

### Only particular residues at position 170 rendered Hog1 intrinsically active

**Figure 5:**
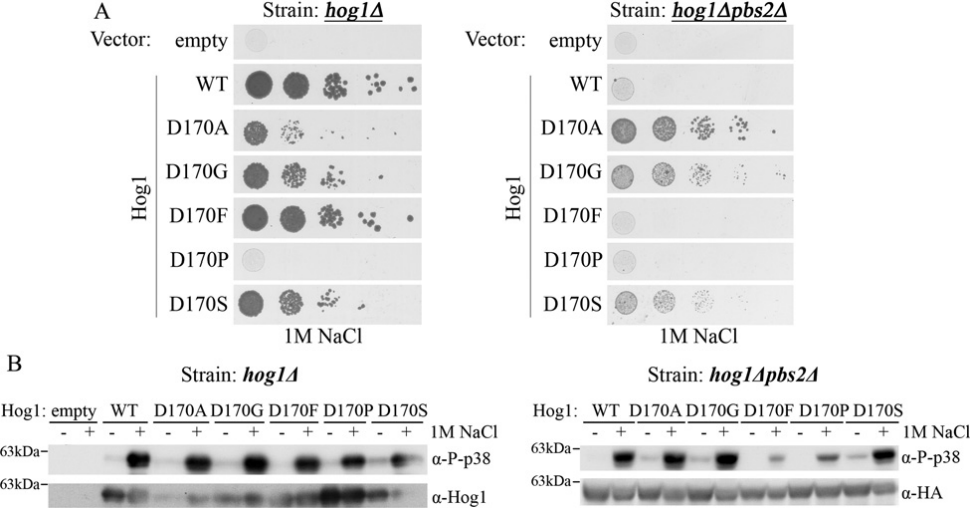
Only specific residues at position 170 render Hog1 intrinsically active (**A**) *hog1Δ* cells (left panel) and *hog1Δpbs2Δ* cells (right panel) expressing Hog1 molecules with the indicated residues at position 170 were plated in five dilutions on plates containing YPD supplemented with 1 M NaCl. (**B**) Western blot analysis with the indicated antibodies was performed on protein lysates prepared from *hog1Δ* cells (left panel) or from *hog1Δpbs2Δ* cells (right panel), expressing the indicated Hog1 molecules. Cells were grown to logarithmic phase on YNB (-URA) medium, washed and incubated in YPD supplemented with 1 M NaCl for 10 min prior to lysis. α-Phospho-p38 antibody was used to determine the phosphorylation of the TGY motif of Hog1. α-HA or α-Hog1 antibodies were used for determination of the steady-state levels of expressed Hog1 proteins.

Conversion of Asp^170^, which resides within the activation loop, to Ala, rendered Hog1 intrinsically active [34]. In the crystal structures of non-active and active p38α (Figure 2), Asp^176^ (the equivalent of Asp^170^ of Hog1) does not form any intra-molecular interaction, faces the solvent and is clearly not involved in the network of αC-helix–αL16-helix interactions. It implies that the D170A mutation uses a totally different mechanism for rendering Hog1 intrinsically active. If the D170A mutation also breaks some critical suppressor of autophosphorylation perhaps at this site too any modification could evoke intrinsic activity. To test this idea we substituted Asp^170^ with Gly, Phe, Pro or Ser. It was observed that only Hog1^D170A^ rescued *hog1∆pbs2∆* cells efficiently. Other small residues at position 170, Gly and Ser, also rendered Hog1 independent of Pbs2, but less efficient than Ala (Figure 5A). The large hydrophobic Phe at position 170 did not render Hog1 Pbs2-independent. Importantly, Hog1^D170F^ was able to rescue *hog1∆* cells suggesting that it is an intact protein that can be activated properly by Pbs2 (Figure 5A, left panel). Hog1^D170P^ was not able to rescue either *hog1∆* or *hog1∆pbs2∆* cells (Figure 5A). All variants, including Hog1^D170F^ and Hog1^D170P^, were phosphorylated to different levels in *hog1∆pbs2∆* cells in response to osmotic pressure (Figure 5B).

### Unlike the case of Hog1, in the mammalian MAPK p38α, only mutations in Phe^327^ render the kinase intrinsically active

Based on the sequence conservation between Hog1 and p38, in previous studies we inserted mutations, equivalent to those that render Hog1 intrinsically active, to p38 isoforms. We found that p38α^F327L^ and p38α^F327S^ (Phe^327^ is equivalent to Phe^322^ of Hog1) acquired a significant intrinsic activity ([26,28,29]; summarized in Table 1). In addition, several p38α molecules mutated at Tyr^323^ (equivalent to Phe^318^ of Hog1) exhibited low intrinsic activity *in vitro* [30]. Conversely, p38α^Y69H^, p38α^W337R^, p38α^A320T^ and a series of 11 mutants, carrying mutations at the 323 position of p38α, did not demonstrate intrinsic activity in these assays [26,28–30]. Notably, mutating Asp^176^ of p38α, which is equivalent to Asp^170^ of Hog1, which resides outside the ‘hydrophobic core’, to Ala, did render the p38α intrinsically active as tested *in vitro* ([26,29]; Table 1).

**Figure 6:**
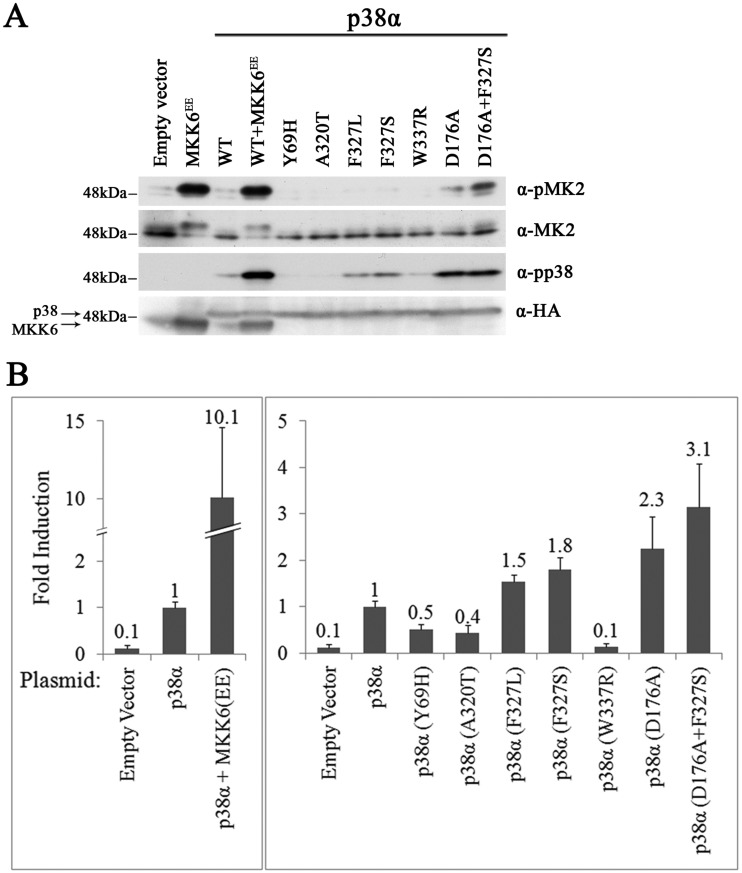
Only specific mutations render p38α spontaneously active in HEK293 cells (**A**) pCDNA3 vectors carrying HA-tagged cDNAs encoding the indicated p38α proteins were introduced into HEK293 cells. Forty-eight hours post transfection, cells were harvested and subjected to a western blot analysis using the indicated antibodies. Lysate of cells transfected with an empty vector was used as a negative control and lysate of cells transfected with vectors expressing p38α^WT^ and MKK6^EE^ was used as a positive control. (**B**) The indicated pCDNA3 expression vectors were introduced into HEK293T cells together with the AP-1-luciferase reporter gene. A vector that constitutively expresses *Renilla*-luciferase was also included in all transfections. Luciferase activities were measured 48 h post transfection by the Dual Luciferase System (Promega). Results are the average of three independent biological experiments, each performed in triplicates. The numbers presented are fold induction of luciferase activity using the activity measured in cells expressing p38α^WT^ as 1. Error bars denote standard errors.

While the p38 mutants were tested only *in vitro*, the Hog1 mutants were analysed in both a biological assay (rescuing *hog1∆* and *pbs2∆* cells from osmotic stress) and in a biochemical assay (IP kinase assay; [33–35]). The biological assay (rescue of *pbs2∆* cells from osmotic stress) seems to be very sensitive. This assay ensures that even if a mutant acquired only weak intrinsic activity, which is not necessarily reflected in a high phosphorylation state or catalytic capabilities, it would still be identified as intrinsically active. Given the observations in yeast, we wondered whether this could be the case in p38 as well, namely, mutants that do not show increased phosphorylation levels or intrinsic catalytic activity *in vitro* could still be in fact intrinsically active to some degree, but this activity would be observed only in living cells. To check this hypothesis, a battery of the p38α mutants, including those that did not show activity *in vitro*, was tested in mammalian cells. p38α^Y69H^, p38α^A320T^, p38α^F327L^, p38α^F327S^ and p38α^W337R^ were expressed in the human cell line HEK293T. The intrinsically active variants p38α^D176A^ and p38α^D176A+F327S^ served as positive controls. Two assays that reflect p38α downstream effects were applied: first, the monitoring of the phosphorylation status of MAPKAPK2 (MK2), a direct p38 substrate (Figure 6A); second, activation of an AP-1-luciferase reporter gene (Figure 6B). Both assays showed that mutants that were not intrinsically active *in vitro* were similarly unable to induce MK2 phosphorylation or AP-1-luciferase activity in HEK293 cells (Figure 6). Thus, most modifications of the ‘hydrophobic core’ of p38α did not render the molecule spontaneously active *in vitro* or in cell culture.

### Mutations in the ‘hydrophobic core’ of p38β, p38γ and p38δ (except p38δ^F324S^) did not render the kinases capable of spontaneous autophosphorylation

In previous studies we examined the effect on other p38 isoforms of some mutations equivalent to those that render Hog1 intrinsically active. We found that only p38δ^F324S^ acquired a significant intrinsic activity [28,29], whereas, p38β^Y323S^, p38β^V327L^, p38γ^F330S^, p38δ^F324L^, p38δ^L328S^ and p38δ^W338R^ did not manifest any intrinsic activity [26,28–30]. Conversely, mutations outside the ‘hydrophobic core’, in residues equivalent to Asp^170^ in Hog1, namely D176A in p38β and D179A in p38γ, did render these MAP kinases intrinsically active ([26,28,29]; Table 1). We now further mutated p38γ at its ‘hydrophobic core’ and produced p38γ^Y72H^, p38γ^Y326S^, p38γ^Y326A^, p38γ^Y326L^, p38γ^Y326T^, p38γ^F330T^, p38γ^F330R^, p38γ^F330A^, p38γ^F330P^ and p38γ^F330L^. We also mutated p38δ’s Phe^324^ residue, producing p38δ^F324A^, p38δ^F324E^, p38δ^F324P^, p38δ^F324R^, p38δ^F324T^ and p38δ^F324Y^. All mutants were tested as purified recombinant proteins *in vitro* and showed no spontaneous activity (summarized in Table 3). Some new mutations were also inserted into p38β, which is unique in the family of MAPKs as it possesses a spontaneous autophosphorylation capability [16]. Previously we reported two mutations at the p38β’s ‘hydrophobic core’; V327L, which did not influence kinase activity, and V327S, which resulted in slightly increased intrinsic activity [28,29]. In p38α, which is 73.6% identical and 86.8% similar to p38β, equivalent mutations, F327L and F327S, rendered it intrinsically active [26,29]. Of the residues that form the ‘hydrophobic core’, all are identical in p38α and p38β, except for position 327, which is occupied by Val in p38β and by Phe in p38α. We postulated that this difference might contribute to the spontaneous autophosphorylation activity of p38β in that the valine residue, which is smaller than phenylalanine, makes the ‘hydrophobic core’ of p38β weaker than that of p38α thus contributing to the intrinsic activity of the former. However, changing Val^327^ to Phe (making the ‘hydrophobic core’ of p38β very similar to that of p38α) did not eliminate the intrinsic activity of p38β. Also, replacing Phe^327^ of p38α with Val did not render p38α intrinsically active (Table 3). Thus, a phenylalanine at this residue is not sufficient to induce a ‘closed’ conformation and to suppress autophosphorylation. In contrast with the situation in p38, changing Phe^322^ of Hog1 to Val did render the protein intrinsically active (Figure 3C), thus strengthening the notion that the ‘hydrophobic core’ of Hog1 is prone to activation by mutagenesis whereas the ‘hydrophobic core’ of p38 is not.

### Mutating the equivalent hydrophobic residues of JNKs did not render it intrinsically active

The interactions between residues in the L16 and αC-helix exist in all MAPKs [41]. The results above show that any mutation in the ‘hydrophobic core’ of Hog1 and some mutations in that of p38α render the protein capable of autoactivation via autophosphorylation. Some of the hydrophobic residues that form the core are conserved in ERKs and JNKs. Like Hog1 and p38s, the Erk1 and Erk2 MAPKs possess an occluded autophosphorylation capability as suggested by the strong autophosphorylation activity shown by some Erk mutants [24,25,31,42]. As we have already reported, mutations that rendered Hog1 and p38 intrinsically active are not relevant for evoking spontaneous autophosphorylation in Erk2 (see Table 1 in ref. [31]). JNK proteins were not reported to possess an autophosphorylation capability and no point mutation that renders them intrinsically active was reported. Nonetheless, one splicing variant, JNK2α2 was reported to autophosphorylate spontaneously *in vitro* [17], suggesting that the JNK family does possess an autophosphorylation capability. To assess whether mutating the ‘hydrophobic core’ of JNK1 would render it spontaneously autophosphorylatable we prepared JNK1^Y71H^, JNK1^L342Q^, JNK1^W352R^, JNK1^I337S^, JNK1^P338Y^ and JNK1^Y71H+W352R^, and assayed them as recombinant proteins. None of the mutants showed any increase in basal, MKK7-independent, activity when tested with c-Jun as a substrate and did not exhibit any autophosphorylation capabilities (Table 3). These results are not entirely surprising because the organization of the core differs in p38s, ERKs and JNKs [43,44].

### Modelling of the Hog1 structure revealed that it may not possess the salt bridge between αC-helix and αL16-helix that exists in p38α

**Figure 7:**
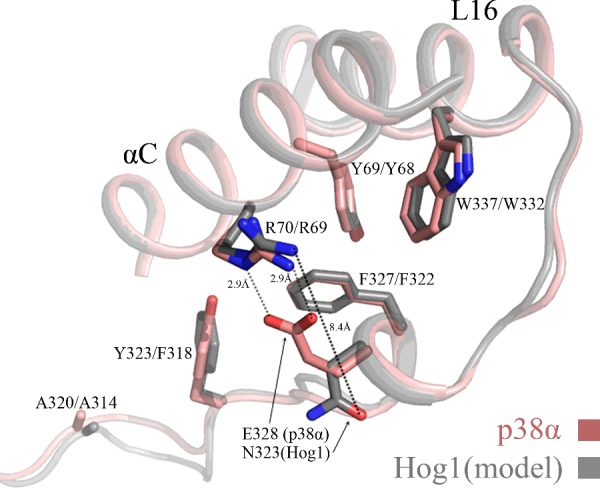
A model of the ‘hydrophobic core’ of Hog1 discloses a more ‘open’ conformation than that of the core of p38α Superimposition of the crystal structure of p38α (pink backbone) and the model of the Hog1 structure (grey backbone) are shown. The crystal structure of non-phosphorylated form of p38α (PDB 1P38) was used as a template for modelling the Hog1 structure, using SWISS-MODEL Workspace (swissmodel.expasy.org/workspace/). Note that the side chains of Arg^70^ and Glu^328^ in p38α form a salt bridge, whereas the side chains of Arg^69^ and Asn^323^ in Hog1 are oriented away from each other.

Although the residues that generate the ‘hydrophobic core’ are highly conserved between Hog1 and p38α, most substitutions of the ‘hydrophobic core’ residues of Hog1 rendered the kinase intrinsically active (Figures 3 and 4, Tables 1 and 3), whereas only a few mutations rendered p38α intrinsically active [(26,28–30]; and Figure 6 and Table 1). What could be the structural explanation for the permissiveness of Hog1’s ‘hydrophobic core’–or conversely the stubbornness of that of p38α–for activating mutations? In an attempt to address this question we modelled a structure of Hog1, based on the available crystal structure of p38α, using SWISS-MODEL Workspace [45,46]. A comparison of the Hog1 model to the crystal structure of p38α revealed a single significant difference in the region of the L16 and αC-helix interactions, at position 328 (p38α numeration). This position is occupied by Asn (Asn^323^) in Hog1, but by Glu (Glu^328^) in p38α (Figure 7). In the model of the non-phosphorylated non-active form of p38α, Glu^328^ interacts with Arg^70^ from the αC-helix, stabilizing the L16–αC-helix interactions [38]. Upon dual phosphorylation Phe^327^ and Glu^328^ are re-oriented away from Arg^70^ that forms instead interactions with phospho-Thr^180^ ([38,39]; and Figure 2). Strikingly, in Hog1, Asn^323^ is oriented away from the ‘hydrophobic core’, so that it resides 8.4 Å (1 Å=0.1 nm) away from Arg^69^. It could be that this orientation of Asn^323^ in Hog1, which leaves the ‘hydrophobic core’ partially ‘open’, in contrast with the ‘closed’ orientation of the Glu^328^ in p38α that keeps the core tighter, makes the Hog1’s ‘hydrophobic core’ less tight and more permissive for activating mutations. In summary, the comparison between the Hog1 model and the crystal structures of active and inactive p38α raises the possibility that Glu^328^ of p38α, via its interaction with Arg^70^, acts as an extra ‘locker’ that secures the ‘hydrophobic core’, preventing an easy opening. These interactions do not exist in Hog1, which possesses an Asn at the equivalent position.

### Mutations in Glu^328^ do not provide p38α with an increased autophosphorylation capability

**Figure 8:**
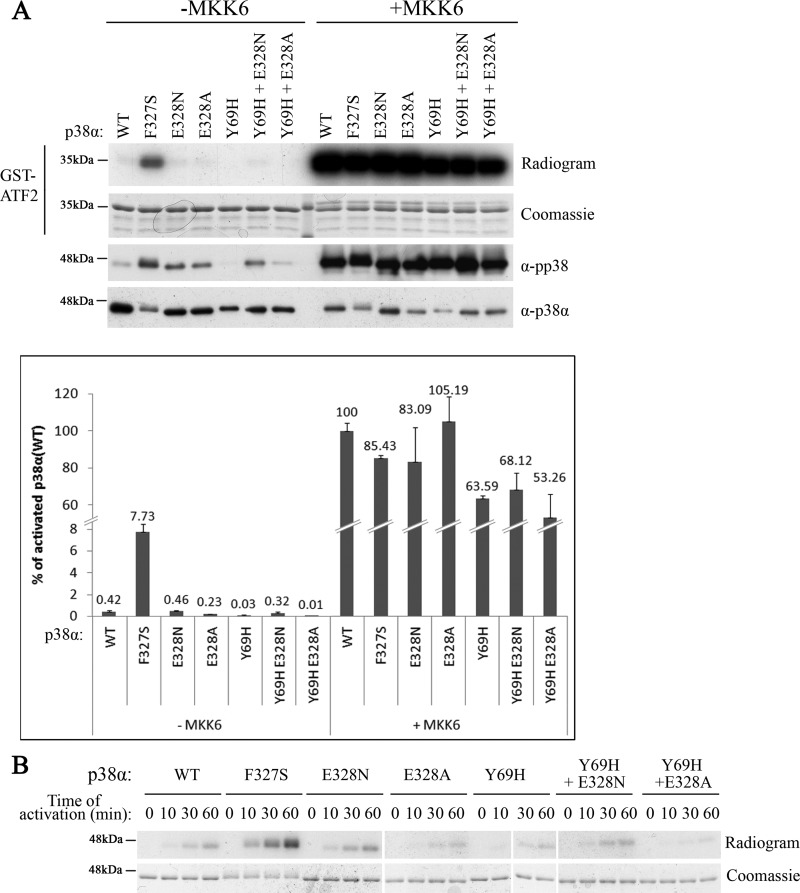
Mutations in the Glu^328^ residue of p38α did not render the protein catalytically active, although induced its autophosphorylation activity (**A**) *In vitro* kinase assay was performed with the indicated p38 proteins with GST-ATF2 as a substrate. Activity is shown in graphs, expressed as percentage of the activity of MKK6-activated wild type p38α (100%), and in autoradiograms (see Materials and methods). The graph shows average results of quantitative measurements of triplicates (see Materials and methods) of the same experiment shown in the autoradiogram. Error bars denote standard deviations. Experiment was performed three times with essentially the same results. One hundred nanograms of each protein was also analysed by western blot with the indicated antibodies. (**B**) Autophosphorylation of the p38α variants was tested by incubating the proteins in a kinase assay mixture with [γ-^32^P]ATP and no other substrate. Samples were removed from the assay at the indicated time points and subjected to SDS/PAGE. Coomassie Brilliant Blue staining verified the amount of p38α protein in each lane.

Given the above structural analysis we tested whether it may be possible to make the ‘hydrophobic core’ of p38α more ‘open’, or more similar to that of Hog1. We converted Glu^328^ of p38α^WT^ to Asn or Ala and also combined these mutations with the Y69H mutation, a mutation that rendered Hog1, but not p38α, intrinsically active (Figures 3 and 6 respectively, Table 1); [26,29,34]. The idea was that perhaps the Y69H mutation would render p38α molecules intrinsically active after all, but only if their ‘hydrophobic core’ was more ‘open’, as it is in Hog1 (i.e. in p38α molecules in which Glu^328^ is converted to Asn). We found, however, that although recombinant purified p38α^E328N^, p38α^E328A^, p38α^E328N+Y69H^ and p38α^E328A+Y69H^ proteins were phosphorylated at somewhat higher levels than p38α^WT^ (Figure 8A), their autophosphorylation activity was low, similar to that of p38α^WT^ (Figure 8B). Accordingly, in an *in vitro* kinase assay with GST-ATF2 as a substrate, the mutants showed no intrinsic activity whatsoever and behaved just like p38α^WT^ (see the radiogram and the graph in Figure 8A).

Thus, it seems that changing the 328 position of p38α to Asn, in an attempt to make its ‘hydrophobic core’ more similar to that of Hog1, did not make the proteins more permissive for activation by mutations. We believe that this particular change was not sufficient to re-orient the conformation and to make it more Hog1-like and more ‘open’ for activating mutations and that other mutations, which are difficult to predict, might achieve this goal.

### Mutations in Asn^323^ of Hog1 weakened the intrinsic activity of Hog1^Y68H^

**Figure 9:**
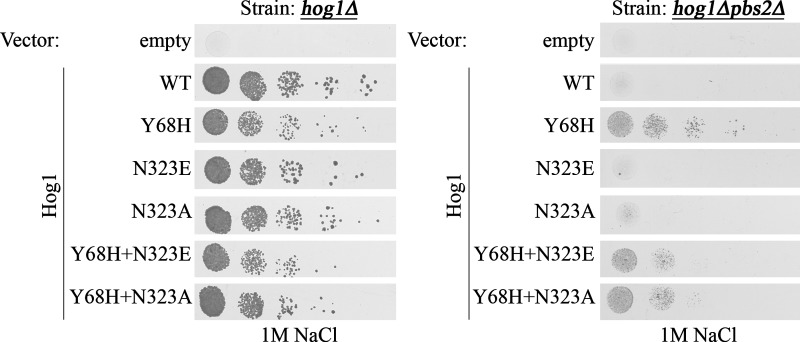
Mutations inserted at position 323 of Hog1, replacing the Asn^323^, in combination with the Y68H mutation caused reduced intrinsic activity in yeast cells *hog1Δ* cells (left panel) and *hog1Δpbs2Δ* cells (right panel), expressing Hog1 molecules carrying the indicated point mutation in ‘hydrophobic core’ residues were plated in five dilutions on plates containing YPD supplemented with 1 M NaCl.

We also tested whether changing the Hog1’s 323 position to Glu would make it more p38α-like and less permissive for activation. We thus prepared *HOG1^N323E^*, *HOG1^N323A^*, *HOG1^Y68H+N323E^* and *HOG1^Y68H+N323A^* genes and expressed them in *hog1∆* and *hog1∆pbs2∆* cells. In the presence of Pbs2 (i.e. in *hog1∆* cells), all mutants grew normally under osmotic pressure (Figure 9, left panel). When expressed in *hog1∆pbs2∆* cells, Hog1^Y68H+N323E^ and Hog1^Y68H+N323A^ could rescue the cells from high osmotic pressure, but less efficiently than Hog1^Y68H^ (Figure 9, right panel).

We surmise that, as in the case of the attempt to make p38α more similar to Hog1 (Figure 8) a single mutation might not be sufficient to stabilize the ‘hydrophobic core’ of Hog1 in a conformation that is very similar to that of p38α.

## DISCUSSION

This study analysed a structural element that seems to play a role in restraining the spontaneous autophosphorylation capability of Hog1 and p38s. This element, termed a ‘hydrophobic core’, does not exist in other EPKs, most of which autophosphorylate spontaneously. The core is structurally defined and precise in p38α, but is only partially conserved in JNKs and ERKs. It does not seem to be involved in regulating autophosphorylation in these MAPKs. αC-helix–L16 interactions were noticed in the crystal structures of Erk2 and p38α, mainly in the structures of the active conformations [38,40,43]. The importance of this core was disclosed when an unbiased genetic screen for intrinsically active variants of Hog1 resulted in a mutation in each of the residues of the core [34]. In T-cells, phosphorylation of Tyr^323^ of p38α, one of the residues forming the ‘hydrophobic core’, induces autophosphorylation at the activation loop [21]. It could be assumed that, unlike the non-phosphorylated tyrosine 323, the phosphorylated Tyr^323^ cannot form hydrophobic interactions. This assumption strongly supports the notion that disrupting the core is sufficient to impose autophosphorylation at the activation loop. Phosphorylation of the Tyr^323^ in T-cells suggests that disrupting the core is utilized as a means to activate p38s under physiological conditions.

Modifying the αC-helix–L16 interactions does not affect activity of MAP kinases of the Erk sub-family (see Table 1 in Ref. [31]). Mutations that do render Erk molecules spontaneously autophosphorylatable do not reside in the L16 domain nor in residues that interact with it [24,25,31]. In addition, no intrinsically active mutants have been identified so far for JNK isoforms, and consequently it is not known whether a point mutation in the ‘hydrophobic core’ could render it intrinsically active. In any case, the JNK1 mutants that were tested here, carrying equivalent mutations to those that rendered p38α intrinsically active, did not affect its activity. It seems that the function of the ‘hydrophobic core’ as a ‘locker’ of autophosphorylation may be specific to the Hog1/p38 family.

Following dual phosphorylation, MAPKs acquire an active conformation that is essentially similar to that of other EPKs [47]. In Erk2, for example, inter-lobe reorientation is stabilized by eight interactions (hydrogen bonds and salt bridges) between the residues of the L16, αC-helix and phospho-Thr^183^ [43]. Those new contacts between L16 and the activation loop result in formation of 3/10 helix at the L16 [43]. In p38α, a 3/10 helix exists in the L16 of the non-phosphorylated protein, and it undergoes some unwinding following activation [39,40]. In particular, Phe^327^, which resides at the centre of the 3/10 helix, is significantly re-oriented following dual phosphorylation, breaking hydrophobic interactions with Tyr^69^ and Trp^337^ [(39]; Figure 2). Re-conformation of the ‘hydrophobic core’ is thus associated not only with autophosphorylation of p38α, but also with its MKK6-dependent activation.

We have now shown that not only the particular mutations identified in the screen for intrinsically active Hog1 molecules, but actually any mutation that alters the ‘hydrophobic core’ interactions, renders the resulting Hog1 mutants autophosphorylatable and intrinsically active. These results suggest that the core, rather than being stable and anchored, is fragile, so that any modification impairs its function as a suppressor of spontaneous autophosphorylation.

The ‘hydrophobic core’ is not the only structural element controlling autophosphorylation of Hog1 and p38α. We have previously reported that the long C-terminal tail of Hog1 (Hog1 is 80 amino acids longer than p38α) is an inhibitory component, and that removing it creates intrinsically active molecules [32]. Accordingly, a specific point mutation in this ‘tail’, N391D, renders the kinase intrinsically active [34]. A unique sequence of five residues within the C-terminal tail (in the region that exists in p38α too) is critical for executing autophosphorylation in both Hog1 and p38α [32]. Finally, a short sequence within the MKI seems to be involved in controlling autophosphorylation of p38α and p38β. It is a 13-amino-acid-long fragment that includes a few residues from the αG-helix and residues from the MKI [16]. It is not clear whether and how this fragment, the C-terminal 5-amino-acid fragment and the ‘hydrophobic core’ are related. Perhaps each of these elements controls autophosphorylation via an independent and exclusive mechanism. As all these elements are located within MAPK-specific structural domains, i.e. the MKI and the C-terminal extension, it may be that suppressing spontaneous autophosphorylation was the driving force for the appearance in evolution of these structural elements. These MAPK-specific structural elements do not hermetically shut-off the autophosphorylation of Hog1/p38s, as modifications of each of them by itself is sufficient to render the kinases spontaneously autophosphorylatable. The fact that there are biochemical pathways that activate p38s, and perhaps Hog1 too, not via the canonical mitogen-activated protein kinase kinase kinase (MKKK)-MKK cascade, but via induced autophosphorylation [18,21,22,32], may explain why evolution kept the suppressing elements somewhat loose and why they are readily de-repressed.

The existence of the salt bridge between Arg^70^ and Glu^328^ in p38α, and its absence from the Hog1 model seems to be the only major structural difference between the ‘hydrophobic cores’ of the two proteins. Only when the crystal structure of Hog1 will be available and when the fine details of the structure–function relationships of both proteins will be revealed it will be possible to be unambiguously conclusive on whether the salt bridge is responsible for p38α being less prone to activation by mutating the ‘hydrophobic core’. Other valid explanations that are not apparent in the model and structure could be different oligomeric state of the two proteins or small differences in the conformation of the ‘hydrophobic core’.

Perhaps because spontaneous autophosphorylation is not efficiently suppressed by inherent elements, cellular regulators that further reinforce this suppression were developed. This is concluded from several observations. First, p38β and JNK2α2, which show spontaneous autophosphorylation *in vitro*, are not spontaneously active in living cells [16,17]. Second, none of the isoforms of p38 and Erk is spontaneously active in mammalian cells, but they are spontaneously autophosphorylated and catalytically active when expressed in yeast cells [48]. It seems that although many aspects of signal transduction are similar in yeast and mammals [49], yeast cells probably lack the cellular components that suppress spontaneous autophosphorylation of the mammalian MAPKs. These mammalian-specific cellular suppressors of autophosphorylation may have co-evolved with mechanism that de-represses them, such as TAB1 binding [19].

For p38α to become autophosphorylated, the substrates of this reaction Thr^180^ and Tyr^182^, need to be activated so that they can nucleophilically attack ATP. Activation of the substrates of p38α and Hog1 is performed by Asp^150^ and Asp^144^ respectively. Perhaps the major general role of the ‘hydrophobic core’, the C-terminal extension and the 13-amino-acids fragment of the MKI, is to prevent access of Thr^180^ and Tyr^182^ to Asp^150^.

## CONCLUSIONS

Unlike most EPKs, MAPKs do not undergo spontaneous autophosphorylation, but instead are activated by dedicated kinases, known as MKKs. One of the critical motifs that functions in Hog1 and p38s to prevent autophosphorylation seems to be a ‘hydrophobic core’, formed by hydrophobic interactions between the αC-helix, which is highly conserved in all EPKs, and the L16, a MAPK-specific element. In Erks and JNKs autophosphorylation is suppressed by other structural elements. In the yeast MAPK Hog1, any mutation in residues that form the core renders the kinase intrinsically active by de-repressing its autophosphorylation capability. Most residues that form the core are conserved in the mammalian p38s, but in these kinases only a few mutations evoke autophosphorylation. It seems that in the course of evolution specific structural elements developed to suppress spontaneous autophosphorylation of Hog1/p38s, but allowed easy de-repression of this activity. This was necessary in order to preserve the ability to activate Hog1 and p38, under certain physiological conditions by induced autophosphorylation. In mammalian p38s de-repression of autophosphorylation occurs less readily than in Hog1, probably due to the appearance of an additional salt bridge between the L16 and the αC-helix. We propose that because of the dramatic effect of MAPKs on the fate of the cell, the activity of MAPKs must be tightly controlled and basal activity cannot be allowed. The basic capability of autophosphorylation was maintained in the course of evolution, but concealed under unique structural motifs such as the ‘hydrophobic core’. Specific native mechanisms unmask this autophosphorylation capability in part by disrupting these motifs. In the mammalian p38s, suppression of autophosphorylation is more sufficient than in the yeast Hog1, and specific enzymatic mechanisms have developed to de-repress it.

## Abbreviations

EPK: eukaryotic protein kinase
Erk: extracellular signal regulated kinase
Hog1: high osmolarity glycerol
JNK: c-Jun N-terminal kinase
MAPK: mitogen-activated protein kinase
MKI: MAPK insert
MKK: mitogen-activated protein kinase kinase
MKKK: mitogen-activated protein kinase kinase kinase

## References

[1] Taylor S.S., Yang J., Wu J., Haste N.M., Radzio-Andzelm E., Anand G. (2004). PKA: a portrait of protein kinase dynamics. Biochim. Biophys. Acta.

[2] Meharena H.S., Chang P., Keshwani M.M., Oruganty K., Nene A.K., Kannan N., Taylor S.S., Kornev A.P. (2013). Deciphering the structural basis of eukaryotic protein kinase regulation. PLoS Biol..

[3] Oruganty K., Kannan N. (2012). Design principles underpinning the regulatory diversity of protein kinases. Philos. Trans. R. Soc. Lond. B Biol. Sci..

[4] Shi Z., Resing K.A., Ahn N.G. (2006). Networks for the allosteric control of protein kinases. Curr. Opin. Struct. Biol..

[5] Johnson L.N., Noble M.E., Owen D.J. (1996). Active and inactive protein kinases: structural basis for regulation. Cell..

[6] Huse M., Kuriyan J. (2002). The conformational plasticity of protein kinases. Cell..

[7] Nolen B., Taylor S., Ghosh G. (2004). Regulation of protein kinases; controlling activity through activation segment conformation. Mol. Cell.

[8] Pike A.C., Rellos P., Niesen F.H., Turnbull A., Oliver A.W., Parker S.A., Turk B.E., Pearl L.H., Knapp S. (2008). Activation segment dimerization: a mechanism for kinase autophosphorylation of non-consensus sites. EMBO J..

[9] Lochhead P.A. (2009). Protein kinase activation loop autophosphorylation in cis: overcoming a Catch-22 situation. Sci. Signal..

[10] Taylor S.S., Kornev A.P. (2011). Protein kinases: evolution of dynamic regulatory proteins. Trends Biochem. Sci..

[11] Lolli G., Johnson L.N. (2005). CAK-cyclin-dependent activating kinase: a key kinase in cell cycle control and a target for drugs?. Cell Cycle.

[12] Chen Z., Gibson T.B., Robinson F., Silvestro L., Pearson G., Xu B., Wright A., Vanderbilt C., Cobb M.H. (2001). MAP kinases. Chem. Rev..

[13] Kyriakis J.M., Avruch J. (2001). Mammalian mitogen-activated protein kinase signal transduction pathways activated by stress and inflammation. Physiol. Rev..

[14] Marshall C.J. (1994). MAP kinase kinase kinase, MAP kinase kinase and MAP kinase. Curr. Opin. Genet. Dev..

[15] Taylor S.S., Keshwani M.M., Steichen J.M., Kornev A.P. (2012). Evolution of the eukaryotic protein kinases as dynamic molecular switches. Philos. Trans. R. Soc. Lond. B Biol. Sci..

[16] Beenstock J., Ben-Yehuda S., Melamed D., Admon A., Livnah O., Ahn N.G., Engelberg D. (2014). The p38beta mitogen-activated protein kinase possesses an intrinsic autophosphorylation activity, generated by a short region composed of the alpha-G helix and MAPK insert. J. Biol. Chem..

[17] Cui J., Holgado-Madruga M., Su W., Tsuiki H., Wedegaertner P., Wong A.J. (2005). Identification of a specific domain responsible for JNK2alpha2 autophosphorylation. J. Biol. Chem..

[18] Ge B., Gram H., Di Padova F., Huang B., New L., Ulevitch R.J., Luo Y., Han J. (2002). MAPKK-independent activation of p38alpha mediated by TAB1-dependent autophosphorylation of p38alpha. Science.

[19] De Nicola G.F., Martin E.D., Chaikuad A., Bassi R., Clark J., Martino L., Verma S., Sicard P., Tata R., Atkinson R.A. (2013). Mechanism and consequence of the autoactivation of p38alpha mitogen-activated protein kinase promoted by TAB1. Nat. Struct. Mol. Biol..

[20] Kang Y.J., Seit-Nebi A., Davis R.J., Han J. (2006). Multiple activation mechanisms of p38alpha mitogen-activated protein kinase. J. Biol. Chem..

[21] Salvador J.M., Mittelstadt P.R., Guszczynski T., Copeland T.D., Yamaguchi H., Appella E., Fornace A.J., Ashwell J.D. (2005). Alternative p38 activation pathway mediated by T cell receptor-proximal tyrosine kinases. Nat. Immunol..

[22] Mittelstadt P.R., Salvador J.M., Fornace A.J., Ashwell J.D. (2005). Activating p38 MAPK: new tricks for an old kinase. Cell Cycle.

[23] Good M., Tang G., Singleton J., Remenyi A., Lim W.A. (2009). The Ste5 scaffold directs mating signaling by catalytically unlocking the Fus3 MAP kinase for activation. Cell..

[24] Emrick M.A., Hoofnagle A.N., Miller A.S., Ten Eyck L.F., Ahn N.G. (2001). Constitutive activation of extracellular signal-regulated kinase 2 by synergistic point mutations. J. Biol. Chem..

[25] Emrick M.A., Lee T., Starkey P.J., Mumby M.C., Resing K.A., Ahn N.G. (2006). The gatekeeper residue controls autoactivation of ERK2 via a pathway of intramolecular connectivity. Proc. Natl. Acad. Sci. U.S.A..

[26] Diskin R., Askari N., Capone R., Engelberg D., Livnah O. (2004). Active mutants of the human p38alpha mitogen-activated protein kinase. J. Biol. Chem..

[27] Askari N., Diskin R., Avitzour M., Yaakov G., Livnah O., Engelberg D. (2006). MAP-quest: could we produce constitutively active variants of MAP kinases?. Mol. Cell Endocrinol..

[28] Avitzour M., Diskin R., Raboy B., Askari N., Engelberg D., Livnah O. (2007). Intrinsically active variants of all human p38 isoforms. FEBS J..

[29] Askari N., Diskin R., Avitzour M., Capone R., Livnah O., Engelberg D. (2007). Hyperactive variants of p38alpha induce, whereas hyperactive variants of p38gamma suppress, activating protein 1-mediated transcription. J. Biol. Chem..

[30] Tzarum N., Diskin R., Engelberg D., Livnah O. (2011). Active mutants of the TCR-mediated p38alpha alternative activation site show changes in the phosphorylation lip and DEF site formation. J. Mol. Biol..

[31] Levin-Salomon V., Kogan K., Ahn N.G., Livnah O., Engelberg D. (2008). Isolation of intrinsically active (MEK-independent) variants of the ERK family of mitogen-activated protein (MAP) kinases. J. Biol. Chem..

[32] Maayan I., Beenstock J., Marbach I., Tabachnick S., Livnah O., Engelberg D. (2012). Osmostress induces autophosphorylation of Hog1 via a C-terminal regulatory region that is conserved in p38alpha. PLoS One.

[33] Bell M., Engelberg D. (2003). Phosphorylation of Tyr-176 of the yeast MAPK Hog1/p38 is not vital for Hog1 biological activity. J. Biol. Chem..

[34] Bell M., Capone R., Pashtan I., Levitzki A., Engelberg D. (2001). Isolation of hyperactive mutants of the MAPK p38/Hog1 that are independent of MAPK kinase activation. J. Biol. Chem..

[35] Yaakov G., Bell M., Hohmann S., Engelberg D. (2003). Combination of two activating mutations in one HOG1 gene forms hyperactive enzymes that induce growth arrest. Mol. Cell Biol..

[36] Hohmann S. (2002). Osmotic stress signaling and osmoadaptation in yeasts. Microbiol. Mol. Biol. Rev..

[37] Saito H., Posas F. (2012). Response to hyperosmotic stress. Genetics.

[38] Sours K.M., Kwok S.C., Rachidi T., Lee T., Ring A., Hoofnagle A.N., Resing K.A., Ahn N.G. (2008). Hydrogen-exchange mass spectrometry reveals activation-induced changes in the conformational mobility of p38alpha MAP kinase. J. Mol. Biol..

[39] Zhang Y.Y., Wu J.W., Wang Z.X. (2011). Mitogen-activated protein kinase (MAPK) phosphatase 3-mediated cross-talk between MAPKs ERK2 and p38alpha. J. Biol. Chem..

[40] Diskin R., Lebendiker M., Engelberg D., Livnah O. (2007). Structures of p38alpha active mutants reveal conformational changes in L16 loop that induce autophosphorylation and activation. J. Mol. Biol..

[41] Nguyen T., Ruan Z., Oruganty K., Kannan N. (2015). Co-conserved MAPK features couple D-domain docking groove to distal allosteric sites via the C-terminal flanking tail. PLoS One.

[42] Smorodinsky-Atias K., Goshen-Lago T., Goldberg-Carp A., Melamed D., Shir A., Mooshayef N., Beenstock J., Karamansha Y., Darlyuk-Saadon I., Livnah O. (2016). Intrinsically active variants of Erk oncogenically transform cells and disclose unexpected autophosphorylation capability that is independent of TEY phosphorylation. Mol. Biol. Cell..

[43] Canagarajah B.J., Khokhlatchev A., Cobb M.H., Goldsmith E.J. (1997). Activation mechanism of the MAP kinase ERK2 by dual phosphorylation. Cell.

[44] Oza V., Ashwell S., Almeida L., Brassil P., Breed J., Deng C., Gero T., Grondine M., Horn C., Ioannidis S. (2012). Discovery of checkpoint kinase inhibitor (S)-5-(3-fluorophenyl)-N-(piperidin-3-yl)-3-ureidothiophene-2-carboxamide (AZD7762) by structure-based design and optimization of thiophenecarboxamide ureas. J. Med. Chem..

[45] Arnold K., Bordoli L., Kopp J., Schwede T. (2006). The SWISS-MODEL workspace: a web-based environment for protein structure homology modelling. Bioinformatics.

[46] Bordoli L., Kiefer F., Arnold K., Benkert P., Battey J., Schwede T. (2009). Protein structure homology modeling using SWISS-MODEL workspace. Nat. Protoc..

[47] Kornev A.P., Haste N.M., Taylor S.S., Eyck L.F. (2006). Surface comparison of active and inactive protein kinases identifies a conserved activation mechanism. Proc. Natl. Acad. Sci. U.S.A..

[48] Levin-Salomon V., Maayan I., Avrahami-Moyal L., Marbach I., Livnah O., Engelberg D. (2009). When expressed in yeast, mammalian mitogen-activated protein kinases lose proper regulation and become spontaneously phosphorylated. Biochem. J..

[49] Engelberg D., Perlman R., Levitzki A. (2014). Transmembrane signaling in Saccharomyces cerevisiae as a model for signaling in metazoans: state of the art after 25 years. Cell Signal..

